# A Contribution to the Pathology of the Endometrium

**Published:** 1908-06

**Authors:** 


**A Contribution to the Pathology of the Endometrium.** By Jessie M. Maccregor, M.D. Pp. 52. Edinburgh: William Green & Sons. 1905.

The observations recorded in this little work will prove of the greatest possible value to the gynæcologist. The pathological changes which take place in the endometrium are classified on a sound basis, and in place of describing the various forms of so-called endometritis, either by their naked-eye appearance or their clinical symptoms, the descriptions are based on careful and accurate microscopic observations of portions of structures removed by the curette. A large number of cases were examined in this way, and the various results tabulated with several very important results.

In the first and perhaps the most important instance the authoress has been able to lay down certain features which indicate the transitional stages between conditions usually regarded as benign and malignant disease. For example, she states that as long as the epithelial cells retain their cylindrical character with an ellipsoid nucleus the condition may be ranked as innocent, even though there may be many nuclei among their bases, and a tendency to break into irregular arrangement. If, on the other hand, the cells lose their cilia, tend to break into polyhedral and spherical shapes, and show irregular growth of well-formed cells with rounded nuclei, the verdict must be that the condition is malignant; and this applies also to those cases in which at any part the cells break through the gland wall into the stroma. In the case where the specimen shows doubtful adenoma, she states that simple multiplication of glands which remain fairly equal in size and regular in colour, even though the stroma is reduced to a few strands, is not necessarily a sign of malignancy. On the other hand, marked increase or irregularity in size of the glands, or papillary invasion of the lumen, is always suspicious. If the epithelium of the papillary ingrowth is young, its nuclei staining deeply, the case is distinctly suspicious, and if in addition there are areas of deeply-staining cells in the stroma the specimen may be regarded as malignant.

She shows the difficulties of diagnosis of early sarcoma of the endometrium, chiefly due to points of similarity between the stroma of the normal endometrium and sarcoma. Every variety of stroma cell, such as those of small round, large fusiform, or branching myxomatous type, can be compared with similar forms of sarcoma cells ; and even the giant-celled type may be found in *post-abortum* cases, where among all shapes and sizes of cells the wandering cells of the syncytium may be found.

The difficulties in the way of acurate diagnosis of sarcoma of the endometrium is thus shown to be very great. As regards inflammatory conditions, and more especially that usually described as uterine catarrh, the following classification is suggested : (1) œdematous degeneration ; (2) non-malignant new growth ; (3) persistent products of pregnancy ; (4) arterio-sclerosis. The authoress makes the very important practical deduction that in this case success in treatment depends on the removal of the endometrium with its leaky capillaries, this last condition being the cause of the hemorrhage which accompanies most of these changes. Hence curetting is the only method of treatment for cases in which decidual products have been retained. In arterio-sclerosis, on the other hand, curetting may be most baneful, and may cause such profuse hemorrhage as to necessitate hysterectomy, since in this case the rigid walled arteries will be laid open. The hemorrhage depends, therefore, not only on the leaky vessels of the endometrium, but also on the condition of the uterine wall and the general circulation ; or in other words, it may be caused either by the leaky and spongy endometrium or by changes in the deeper structures. The former condition is amenable to active operative treatment, but the latter is not.

This brief extract will serve as an indication of the care and attention with which this contribution has been written. It should be read carefully by everyone who practices gynæcology, since it shows very clearly that unless the interpretation of clinical symptoms rests upon an accurate pathological basis harm instead of benefit may result from active treatment indiscriminately applied.

We should like to express our regret for the sad circumstance of the death of the gifted authoress of this volume, from whom in the future much good work might have been expected.

